# Sagittal lip positions in different skeletal malocclusions: a cephalometric analysis

**DOI:** 10.1186/s40510-015-0077-x

**Published:** 2015-05-01

**Authors:** Merina Joshi, Li Peng Wu, Surendra Maharjan, Mukunda Raj Regmi

**Affiliations:** Department of Stomatology, College of Stomatology, Jiamusi University, Street no. 522, Hongqi street, Jiamusi, 154004 Republic of China; Department of Health Services, Teku, Kathmandu, 44600 Nepal

**Keywords:** Northeast Chinese population, Skeletal class, Reference lines, Sagittal, Lip

## Abstract

**Background:**

The objectives of this paper are to (1) study use of soft tissue analyses advocated by Steiner, Ricketts, Burstone, Sushner and Holdway to develop soft tissue cephalometric norms as baseline data for sagittal lip position in Northeast Chinese adult population, (2) compare the sagittal lip positions in different skeletal malocclusions and (3) compare the sagittal lip positions in Northeast Chinese adults with other reported populations.

**Methods:**

Lateral cephalometric radiographs of subjects were taken in natural head position. Radiographs were manually traced and five reference lines - Sushner, Steiner, Burstone, Holdway and Ricketts, were used. The linear distance between the tip of the lips and the five reference lines were measured. Statistical analysis was done using the Statistical Package for Social Sciences (SPSS) 21. Descriptive analysis was done for each variable for each subject. Coefficient of variation between lip positions as assessed by reference lines was determined. *Post hoc* Tukey’s test was used for comparison of the mean cephalometric values of three skeletal malocclusions. The level of significance for the analysis was set at *p* < 0.05.

**Results:**

The findings showed significant difference in the sagittal lip positions in different skeletal malocclusions. There was variation in consistent reference line in each skeletal malocclusion. The S2 line was the most consistent reference line in skeletal class I and class II group. The B line was the most consistent line in skeletal class III. In skeletal class II group, upper lips were the most protrusive and lower lips were retrusive than in skeletal class I and class III groups. In case of skeletal class III group, upper lips were retrusive and lower lips were more protrusive than in skeletal class I and class II groups.

**Conclusions:**

The sagittal lip positions were found to be associated with the skeletal malocclusion pattern. Northeast Chinese population has protrusive upper and lower lip in comparison to Caucasians. Each skeletal malocclusion group showed different preferable reference lines for analysis of sagittal lip position.

**Electronic supplementary material:**

The online version of this article (doi:10.1186/s40510-015-0077-x) contains supplementary material, which is available to authorized users.

## Background

**Figure 1:**
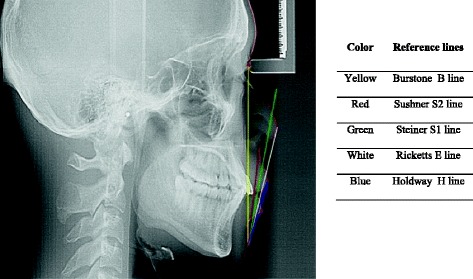
**Lateral cephalometric radiograph representing the five reference lines.**

The soft tissue analysis has always been an integral part of diagnosis and treatment planning. The nose, lip and chin are the major components of soft tissue profile. Various soft tissue analyses have been developed to help clinicians to quantitatively evaluate the facial morphology. Among this, position of lips profoundly alters the choice of treatment. Moreover, orthodontic treatment plan can also alter the lip positions. Lip position has become one of the most important soft tissue analyses as it influences the occlusion, tooth stability and facial aesthetic [1]. The anteroposterior lip position can be evaluated by various reference lines such as Sushner’s S2 line, Steiner’s S1 line, Burrstone’s B line, Ricketts E line and Holdway’s H line (Figure 1).

These normative cephalometric values for soft tissue analyses are based on the studies carried out on Caucasians samples of European-American ancestry (Table 1). Many investigators have proved that there are vast differences among different ethnic and racial groups. They have developed cephalometric standards for different groups and should be treated according to their own characteristics [7]. The adaptation of facial tissues over underlying skeletal discrepancy varies among different races and population. This greatly influences the treatment planning and also the success of treatment. The cephalometric norms reported by these authors may not be suitable to serve as reference values for treatment planning in the Chinese population. Therefore, the objectives of this study are to (1) investigate use of soft tissue analyses advocated by Steiner, Ricketts, Burstone, Sushner and Holdway to develop soft tissue cephalometric norms as baseline data for sagittal lip position in Northeast Chinese adult population for orthodontic diagnosis and treatment planning, (2) compare sagittal lip position in different skeletal malocclusions and (3) compare the sagittal lip position in Northeast Chinese adults with other reported populations.

**Table 1:** **Normal values for the five reference lines [** 1 **-** 6 **]**

**Reference lines**	**Sushner’s S2 line**	**Burstone’s B line**	**Steiner’s S1 line**	**Holdway H line**	**Ricketts E line**
Normal values in mm	Female	3.5 ± 1.4 UL	Lips should touch the reference line	LL should touch the reference or line −1 to +2	3 to 4 UL
					−2 LL
	8.8 UL	2.2 ± 1.6 LL			
	6.7 LL				
	Male				
	10.3 UL				
	7.8 LL				

UL, upper lips; LL, lower lips.

## Methods

This retrospective study comprised of 150 randomly selected patients referred to the Jiamusi University Dental Department. The subjects were selected according to the following criteria:

1. Northeast Chinese population with Northeast Chinese grandparents,
2. Age between 18 and 25 years at the time of cephalometric radiograph,
3. Presence of all permanent teeth except third molars, no anterior or posterior cross bite,
4. No previous history of orthodontic and prosthodontic treatment,
5. No congenital abnormalities or trauma to face and
6. Good lateral cephalometric radiographs.

**Figure 2:**
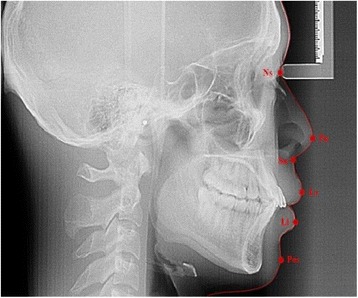
**Cephalometric radiograph showing soft tissue landmarks.**

Consents were obtained from each patient. Lateral cephalometric radiographs were taken in natural head position with the eyes straight ahead, the teeth in centric occlusion and lips in relaxed contact. The same operator took all the lateral cephalometric radiographs. An acetate sheet of 0.003 mm was placed on top X-ray film and traced by 0.5-mm lead pencil. Soft tissue and hard tissue landmarks were traced on acetate sheets (Table 2 and Figure 2). Skeletal malocclusion was classified based upon the ANB (A point, nasion, B point) angle [3,8] and Wits value [9,10] which indicates the positional relationship of the maxilla and mandible Table 3.

**Table 2:** **Cephalometric soft tissue landmarks [** 11 **]**

**Soft tissue landmarks**	**Description**
Soft tissue nasion (Ns)	The point of deepest concavity of the soft tissue contour of the root of the nose
Pronasale (Pn)	The most prominent point of the nose
Subnasale (Sn)	The point where the lower border of the nose meets the outer contour of the upper lip
Labial superius (Ls)	The median point in the upper margin of the upper membranous lip
Labial inferius (Li)	The median point in the lower margin of the lower membranous lip
Soft tissue pogonion (Pos)	The most prominent point on the soft tissue contour of the chin

**Table 3:** **Normal values of ANB angle and Wits appraisal for different skeletal malocclusions**

Skeletal malocclusions	Class I	Class II	Class III
ANB angle	0° to 4°	>4°	<0°
Wits value	0 and −3 mm	≥−1 mm	≤−4 mm

The patients were categorized into three skeletal malocclusions classes and each skeletal malocclusion class having 50 patients. The linear distances between the tips of the lips and the five reference lines were measured using digital vernier caliper. When lips were positioned in front of reference line, they were denoted by positive sign; when behind the reference line, they were denoted by negative sign; and zero when lips were on the reference line.

### Statistical analysis

Statistical analysis was done using the Statistical Package for Social Sciences (SPSS) version 21. Descriptive analysis was carried out for each variable for each subject. Coefficient of variation between lip positions as assessed by reference lines was determined. *Post hoc* Tukey’s test was used for comparison of the mean cephalometric values of the three skeletal malocclusions. The level of significance for the analysis was set at *p* < 0.05.

All the radiographic tracing and measurements were performed by one investigator. To avoid observer bias, each radiograph was coded with a number so that the observer was blinded to the skeletal pattern of the subject. To evaluate the presence of any error with digitization and measurement, 30 randomly selected radiographs were again traced and measured by the same investigator 1 week after the initial measurements.

## Results

Table 4 shows the statistical analysis along with the mean and standard deviation of skeletal class I, class II and class III groups. The coefficient of variation in skeletal class I was the least in relation to S2 line in both the upper lip (UL) (coefficient of variation (CV) = 25.41%) and the lower lip (LL) (CV = 27.897%). The E line showed the greatest variation in both UL and LL (UL = 196.04% and LL = 194.05% of CV). In skeletal class II, S2 line related to both UL (CV = 19.89%) and LL (CV = 34.38%) and had the least CV. The H line showed the greatest variation in LL (477.46%). In both UL and LL, E line showed low variation (UL = 117.44% and LL = 356.86% of CV). The CV in skeletal class III was the least in case of B line related to both UL (CV = 17.76%) and LL (CV = 19.75%). The E line showed greatest variation in both UL and LL (UL = 170.87% and LL = 41.22% of CV).

**Table 4:** **Mean standard deviation and result for** ***post hoc*** **test (Tukey’s HSD) showing the difference between skeletal classes**

	**Class I**	**Class II**	**Class III**
UL to S2 line	11.81^a^(±3.00)	13.75^a^(±2.73)	10.42^a^(±1.95)
LL to S2 line	9.04^a^(±2.52)	8.70^b^(±2.99)	11.42^ab^(±2.30)
UL to B line	6.66^a^(±2.00)	7.48^b^(±2.07)	6.87^c^(±1.22)
LL to B line	5.74^a^(±2.31)	4.85^b^(±2.25)	7.77^ab^(±1.53)
UL to S1 line	4.32^a^(±2.03)	5.35^a^(±1.68)	4.55^b^(±1.20)
LL to S1 line	4.22^a^(±2.32)	3.47^b^(±2.29)	5.83^ab^(±1.29)
LL to H line	1.48^a^(±1.36)	0.49^a^(±2.37)	3.58^a^(±1.01)
UL to E line	1.11^a^(±2.19)	2.14^b^(±2.51)	0.82^b^(±1.40)
LL to E line	1.07^a^(±2.09)	0.75^b^(±2.70)	3.44^ab^(±1.41)

Mean values with same letter (“a” or “b”) in superscript are statistically different (p < 0.05). Mean (±SD), n = 50.

An analysis of variance (ANOVA) test was performed, and it showed all lines are different in all the skeletal malocclusions. *Post hoc* Tukey’s test was used for comparison of the mean cephalometric values of the three skeletal malocclusions. The level of significance for the analysis was set at *p* < 0.05. Table 4 shows mean and standard deviation and result for *post hoc* test (Tukey’s HSD) showing the difference between skeletal classes. Additional data of Post Hoc Tukey Test showing the difference between each skeletal class are shown in Additional file 1: Table S1.

### Sushner’s S2 line

Comparison was done among the skeletal classes to access the sagittal lip position using five reference lines. In skeletal class I group, S2 line showed slightly more protrusive UL (11.81 ± 3.00 mm) and LL (9.042 ± 2.52 mm) than norms given by Sushner (UL 8.8/10.3 mm and LL 6.7 to 7.8 mm). In skeletal class II group, ULs (13.75 ± 2.73 mm) were more protruded and LLs (8.70 ± 2.99 mm) were retrusive than skeletal class I and class III groups. In the case of skeletal class III group, the ULs (10.42 ± 1.95 mm) were retrusive and LLs (11.42 ± 2.30 mm) were protrusive in comparison to class I and class II. There were statistically significant differences between the skeletal classes in S2 to UL. In the case of S2 to LL, there were statistically significant difference between class I and class III and class II and class III.

### Burstone’s B line

When using B line, the UL (6.66 ± 2.00 mm) and the LL (5.74 ± 2.31 mm) in skeletal class I group were protrusive than the normal values given by Burstone. In the case of skeletal class II group, UL (7.48 ± 2.07 mm) were more protrusive and LL (4.85 ± 2.25 mm) were retrusive than skeletal class I and class III groups. Skeletal class III group showed most protruded LL (7.77 ± 1.53 mm) than class I and class II. There were no statistically significant differences between the skeletal classes in case of B line to UL (*p* > 0.05). However, there were statistically significant differences between class I and class III and class II and class III in the case of B line to LL.

### Steiner’s S1 line

In skeletal class I group, S1 line showed protrusive UL (4.32 ± 2.03 mm) and LL (4.22 ± 2.32 mm) than norms given by Steiner. In skeletal class II group, UL (7.03 ± 3.66 mm) were more protrusive and LL (5.77 ± 1.18 mm) were retrusive than in skeletal class I and class III groups. In skeletal class III, the UL (4.55 ± 1.20 mm) were retrusive in comparison to skeletal class II and LL were slightly protrusive (5.83 ± 1.29 mm) in comparison to skeletal class I and class II. There were statistically significant differences between class I and class II in the case of S1 line with UL and between class I and class II in the case of S1 line with LL.

### Ricketts E line

In skeletal class I group, both UL (1.11 ± 2.19 mm) and LL (1.07 ± 2.09 mm) were protrusive than norms provided by Ricketts. In skeletal class II group, LL (2.14 ± 2.51 mm) was more protruded than in skeletal class I and class III groups. In the case of skeletal class III, ULs (0.82 ± 1.40 mm) were retrusive and LLs (3.44 ± 1.41 mm) were protrusive in comparison to class I and class II. There were statistically significant differences between class II and class III in the case of E line with UL and between class II and class III and class I and class II in the case of E line with LL.

### Holdway’s H line

Comparing the lower lip with reference to the H line, it was found that the LL (1.48 ± 1.36 mm) in skeletal class I was protrusive than the norms. LLs in skeletal class III were protrusive than in skeletal class I and class II. There were statistically significant differences between class I and class III and class II and class III.

## Discussion

This study is designed to determine the sagittal lip positions in relation to the five reference lines in three different skeletal malocclusions. The sample included adult subjects of age 18 to 25 years as the majority of facial growth is usually completed by 16 to 17 years of age [12]. The subjects were selected from those individuals who had a lateral cephalometric radiograph taken for diagnosis purposes. The selection criteria for many studies are mostly excellent class I molar in relation with good intercuspation, pleasing facial profiles [13,14]. Assessment based on pleasing profiles and satisfactory occlusion is subjective and introduces biases. Also, they do not represent the randomized representation of the particular population. Hence, it is important to analyse these reference lines in different skeletal malocclusions to determine which reference line is more reliable in each skeletal malocclusion.

Erbay et al. found that soft tissue analysis differs according to population. Every race has its one nose and chin characteristics [15]. Sushner developed his norms for black population. Ricketts norms are applied to Caucasians and not to all ethical and racial groups. Thus, using soft tissue norms of one population would be unreliable in diagnosis and treatment planning for another population. It would be helpful to assess the reliability of these reference lines in Northeast Chinese adult population. This would serve as the baseline data for orthodontic diagnosis and treatment planning.

In this study, five reference lines were used because these reference lines are most frequently used during diagnosis and treatment.

### Consistency of reference lines in different skeletal classes

There was variation in consistency of reference line in each skeletal malocclusion. S2 line had the smallest coefficient of variation and provided the narrowest dispersion in skeletal class I and class II groups. In the case of skeletal class III group, B line had the smallest CV. Hence, S2 line and B line can be considered to be the best reference lines in terms of judging the sagittal lip position in skeletal classes. The possible cause for the S2 line and the B line to be the most consistent lines may be the fact that these lines do not transverse any anatomical landmarks of the nose and also the lines are close to skeletal structure.

E line had the largest CV in both skeletal class I and class III groups and had low CV in skeletal class II. On the other hand, H line was the least reliable in the case of skeletal class II. Therefore, E line and H line can be considered as less reliable to judge sagittal lip position in skeletal malocclusions. From a clinical point of view, if the reference line is located closer anteroposteriorly to the lips, it is convenient to judge the lip position. From this point of view, the E and the H lines are more reliable. The less consistency of the E line and the H line may be because these lines pass through nose and UL, respectively. In the Taiwanese, B line was also the best line in terms of consistency. However, E line is of great value because its anterior location makes it convenient for the clinician to use it, but the B line appears the best from the perspective of the value of reference [16]. Ninety-six Anatolian Turkish adult’s horizontal lip position was analysed cephalometrically using the soft tissue analyses of Steiner, Ricketts, Burstone, Sushner, Holdway and Merrifield. The study indicates that in Anatolian Turkish adults, the UL and the LL were retrusive according to the norms of Steiner and Ricketts. The UL was protrusive and the LL was retrusive according to Sushner line and was within normal range according to Burstone’s B line. The LL was similar to the standard proposed by Holdway [15]. Ricketts norms for lips closely resembled to the value found for the attractive profile [17].

### Comparison of skeletal class I with Caucasian norms and other populations

Our study revealed that both the UL and the LL in skeletal class I were more protrusive than normative values of Ricketts, Burstone, Sushner and Steiner. This is due to the fact that the craniofacial morphology between individuals with typical Chinese and Caucasian ancestry shows significant difference. Chinese samples exhibited significantly protrusive UL and LL than Caucasians and also less obtuse nasolabial angle than Caucasian. The protrusion of LL on the profile of Chinese population was associated with significant labial inclination of lower incisors. Another possible explanation for this difference is may be the Chinese adults have less prominent nasal tips and steeper nasal bridge [18].

In our study, the LL in skeletal class I was 0.92 mm less protrusive than the UL with respect to B line. This can be considered as a consistent result. With respect to S1 line in skeletal class I group, UL and LL were more protrusive. This is in contrast to the result given by Steiner. The UL (4.32 mm) was slightly more protrusive than the LL (4.22 mm).

Additionally, the H line reported for the Northeast Chinese population was protrusive than the normal range. Lew compared H angle between Chinese and Caucasians and found that the Chinese samples had more protrusive lips, a more anteriorly placed maxilla than Caucasian and lips that were not harmonious with the H line [13].

There are few previous cephalometric studies of this kind done on Chinese population. P. Yeong and J. Huggare’s research on craniofacial morphology of Singaporean Chinese found significantly greater protrusion of the UL and LL [19]. Lew also studied the Singaporean Chinese adults with aesthetically pleasing profiles and found Chinese facial profile to be less convex compared to Caucasians, with the maxilla more posteriorly located in Chinese. The UL was more protrusive and at a less obtuse nasolabial angle [13]. He also found that Chinese subjects had UL and LL protrusion of 3.4 mm and 3.5 mm, respectively, with ANB angle to be 2.4° [20]. In one of the studies conducted on the Cantonese Chinese male with pleasing or acceptable profile showed both the UL and the LL protruded beyond the Ricketts’ aesthetic planes and also the lips were more protrusive than those described by Burstone’s B line [14].

Adult Nigerian population showed more protrusive UL and LL than the normative values reported for Caucasians [21]. The Korean population is also reported to have greater degree of UL and LL protrusion when compared to a European American sample [22].

Craniofacial cephalometric analysis of Bangladeshi females had significantly more protruded lip positions when compared with the Caucasian group. When compared with the Japanese females, Bangladeshi females had significantly less protrusion. In the case of males, there was no significant difference in lip protrusion [23] (Table 5). Mean and standard deviation of sagittal lip positions in different populations are shown in Additional file 1: Table S2.

**Table 5:** **Summary of different reported populations**

**Study**	**Sample population**	**Age in years**	**Sample size**	**Sample selection**	**Soft tissue analysis**	**Conclusion**
2012	Nigerian population	18 to 25	100	Class I molar and canine relationship, a symmetrical face	Steiner, Rickets, Burstone, Merrifield and Holdway	More protrusive upper and lower lip as compared to normative values reported for Caucasians
2002	Kwangju sample (Korean population)	18 to 20	60	Normal occlusion, class I molar and canine relationship	Steiner, Rickets, Merrifield and Holdway	Greater degree of lip protrusion in comparison to European-American samples
2004	Singapore Chinese children	Mean 12.5 girls, 12.7 boys	81	Class incisor relationship (British standards institute, 1983)	Rickets E line	Boys had more protrusive lips than Malaysian Chinese and less protrusive lips than Hong Kong Chinese
1992	Chinese adult	18 to 24	72	Harmonious facial profiles with presence of intact dentition, no difference was made between orthodontic treated and non-treated subjects	Legan and Burstone analysis, Holdway analysis	Upper and lower lip not in balance with H line, upper and lower lip were positioned more anteriorly
1972	Males of Kwangtung province origin (Cantonese Chinese)	18 to 33	30	Clinically excellent occlusion, class I molar, pleasing profile	E line and B line	Lips protruded beyond E line
2013	Bangladeshi population	23.2 to 14.6	98	Class I occlusion	B line	Females had more protrude lips compared to Caucasians and less protruded lips than Japanese, males no significant difference between Caucasian and Japanese

The relevant soft tissue analysis and conclusions are only mentioned in brief.

These variation in lip position of different populations reinforce the fact that soft tissue features are specific for each given race and ethnicity. And also this comparison with Caucasian and other population group must be interpreted with caution because of variations in sample sizes and also differences in the population.

### Comparison among different skeletal classes

Overall, we can observe that in the case of skeletal class II group, all the reference lines showed the upper lips to be the most protrusive and the lower lips to be retrusive compared to skeletal class I and class III groups. In the case of skeletal class III group, upper lips were retrusive than in skeletal class I and class II and the lower lips more protrusive than in skeletal class I and class II groups.

Statistically significant correlation was found in case of upper lip with S2 line among all three skeletal classes. In the case of lower lip, H line showed significant statistical differences between all skeletal classes. Upper lip sagittal position can be better assessed by S2 line in different skeletal malocclusions. In the case of lower lip sagittal position, H line is the line of choice. This finding may be due to the fact that S2 line is close to skeletal structure and not influenced by the nose. As the lower lip is closely influenced by the upper lip position, H line can be considered as the best line in assessing lower lip position. In the present study, statistically significant difference for all the reference lines in assessing sagittal lip position among all skeletal classes were not observed. The reason behind could be variation in dentoalveolar structures especially the upper and lower incisors.

Surprisingly, there were no significant differences found between class II and class III groups in the case of E line, and also there was no statistically significant difference between skeletal classes in case of B line when assessing the upper lip. E line is influenced by the nose, but in our study, we did not include assessment of the nose. However, if samples had been based on cephalometric values like large ANB differences, incisor relations or profile assessment, we could have seen significant differences between all the skeletal classes. If soft tissue thickness at various points like nasion, subnasion and pogonian and lip thickness and lip strain were considered during analysis, we could have achieved more precise results.

### Factors affecting lip position

Cephalometric measurements of face in terms of aesthetics can be difficult and misleading due to various factors. Several studies had shown that soft and hard tissue changes are highly correlated. Altemus LA, in his study, found that facial balance and harmony are often in compromised or compensated in relationship with skeletal, dental and soft tissue component of the face [24]. Dental factors, such as the inclination of upper and lower anterior relative to the palatal and mandibular plane, respectively, affects the lip positions. The movement of the cervical point of the upper incisor or the incisor retraction with translator movement greatly influenced changes in the UL position in the horizontal plane [25]. As many of the reference lines used for facial analysis pass through the nose and chin, growth and morphology of the nose and chin would greatly affect the lip position. Ricketts E line should be read in relation to the nose and chin. Ricketts E line is influenced by the growth of the nose. Whereas Steiner’s S1 line eliminates half of the changes in integument profile due to the growth of the nose. Nasal growth is eliminated in H line, B line and Sushner line. The relation of the lip with the B line depends on the thickness of the lip and correct position of lips. When lips are equally thick, B line is more reliable. Both chin and nasal position influence the horizontal lip position [16,26,27]. In Turkish adult, significant differences in soft tissue thickness among skeletal malocclusions were observed for the labrale superius, stomion and labrale inferius sites. Moreover, soft tissue thickness at all sites was greater in men than in women [28].

The two main treatment approaches extraction and non-extraction have been highly controversial issues, and it mainly depends upon the patient profile. This controversy becomes even greater when dealing with borderline cases. When planning treatment for class I cases, the extraction decision mainly depends on lower anterior crowding, lower lip to E line, upper crowding and overjet. These four key orthodontic measurements will possibly vary if we take different populations [29].

There are a number of limitations to our study. First, this study utilized a small sample. Second, the gender differentiation was not done. It is difficult to find detailed information regarding each subjects when doing retrospective study. Third, China having the largest population in the world and also having many sub ethnic groups makes it difficult to collect the sample representing the Chinese population.

The growth and development of soft tissue profile is a result of complex changes within hard and soft tissue. Thus, aging factors had to be considered when evaluating soft tissue profile in adults [30]. Most importantly, it is difficult to make a valuable comparison between findings of our study and others since a limited number of studies have been published on this subject. In the future, we can extend our study to broader geographical area and give quantitative data to represent the norms for each skeletal class.

Conventional cephalometric approach encounters several limitations. Cone-beam computed tomography (CBCT) offers the possibility of accurate localization and quantification of even minor asymmetries without distortion and hence much more precise cephalometric analyses [31,32]. In the future, we can extend our study to a broader geographical area and use the latest CBCT technology to give more precise quantitative data to represent the norms for each skeletal class.

## Conclusions

On the basis of lateral cephalometric records from a sample of 150 Northeast Chinese people examined, the following conclusions can be drawn:

1. UL and LL in skeletal class I group are protrusive than norms on all reference lines.
2. Skeletal class II group have the most protrusive UL and retrusive LL in comparison to skeletal class I and class III on all the reference lines.
3. Skeletal class III group has the most protrusive LL in comparison to skeletal class II and class I on all reference lines.
4. In the case of skeletal class I and class II, S2 line is the line of choice to judge the sagittal position of lips in profile analysis.
5. In the case of skeletal class III, B line is the line of choice to judge the sagittal position of lips in profile analysis.

## Additional file

Additional file 1: Table S1. Comparison of sagittal lip positions between skeletal malocclusions. Analysis by Post Hoc Tukey Test. The level of significance for the analysis was set at p<0.05., p value >0.05 was considered as not significant. *Denotes not significant p value. **Table S2.** Mean and standard deviation of sagittal lip positions of different population reported in our article. Values are in millimeter.

## Abbreviation

B line: Burstone’s line
CV: coefficient of variation
CBCT: cone-beam computed tomography
E line: Ricketts line
H line: Holdway line
LL: lower lip
S1 line: Steiner’s line
S2 line: Sushner’s line
UL: upper lip
