# Supplementary material for: Sagittal lip positions in different skeletal malocclusions: a cephalometric analysis
Source: Prog Orthod. 2015 May 1;16:8. doi: 10.1186/s40510-015-0077-x (PMC4416099; doi:10.1186/s40510-015-0077-x)
Supplement: Additional file 1: Table S1. — Comparison of sagittal lip positions between skeletal malocclusions. Analysis by Post Hoc Tukey Test. The level of significance for the analysis was set at p<0.05., p value >0.05 was considered as not significant. *Denotes not significant p value. Table S2. Mean and standard deviation of sagittal lip positions of different population reported in our article. Values are in millimeter. [file 40510_2015_77_MOESM1_ESM.docx]

**ADDITIONAL FILE**

Journal: Progress in Orthodontics

Title: Sagittal lip positions in different skeletal malocclusions: a cephalometric analysis

Authors: Joshi Merina, Li Peng Wu, Maharjan Surendra, Regmi Mukunda Raj

Manuscript no: PIOR-D-14-00181

**Table S1.** Comparison of sagittal lip positions between skeletal malocclusions. Analysis by Post Hoc Tukey Test. The level of significance for the analysis was set at p<0.05., p value >0.05 was considered as not significant. *Denotes not significant p value

| **Reference lines** | **Lips** |  | **Class I** | **Class II** | **Class III** |
| --- | --- | --- | --- | --- | --- |
| **Sushner S2** | Upper lip | **Class I** |  | 0.003 | 0.049 |
|  |  | **Class II** | 0.003 | 0.000 | 0.000 |
| **Sushner S2** | Lower lip | **Class I** |  | 0.838* | 0.000 |
|  |  | **Class II** | 0.838* |  | 0.000 |
| **Steiner S1** | Upper lip | **Class I** |  | 0.022 | 0.811* |
|  |  | **Class II** | 0.022 |  | 0.101* |
| **Steiner S1** | Lower lip | **Class I** |  | 0.245* | 0.002 |
|  |  | **Class II** | 0.245* |  | 0.000 |
| **Rickets E** | upper lip | **Class I** |  | 0.084* | 0.808* |
|  |  | **Class II** | 0.084* |  | 0.017 |
| **Rickets E** | Lower lip | **Class I** |  | 0.789* | 0.000 |
|  |  | **Class II** | 0.789* |  | 0.000 |
| **Holdway H** | Lower lip | **Class I** |  | 0.028 | 0.000 |
|  |  | **Class II** | 0.028 |  | 0.000 |
| **Burstone B** | Lower lip | **Class I** |  | 0.148* | 0.000 |
|  |  | **Class II** | 0.148* |  | 0.000 |

**Table S2.** Mean and standard deviation of sagittal lip positions of different population reported in our article. Values are in millimeter.

|  | **Nigerian** | **Korean** | | **Singapore Chinese** | | **Chinese adult** | **Cantonese Chinese** | **Bangladeshi** |
| --- | --- | --- | --- | --- | --- | --- | --- | --- |
|  |  | Male | Female | Male | Female |  |  |  |
| **LL to S1 line** | 5.89 ±2.23 | 2.62± 2.17 | 2.60 ±1.74 |  |  |  |  |  |
| **UL to S1 line** | 8.19 ±2.60 | 2.87 ±1.80 | 2.97 ±2.07 |  |  |  |  |  |
| **UL to B line** | 9.84 ±2.02 |  |  |  |  | 7.0 ±1.5 | 3.5 | 5.5± 2.1 |
| **LL to B line** | 10.53± 2.40 |  |  |  |  |  | 2.2 | 5.3± 2.2 |
| **UL to E line** | 3.21 ±2.69 | -0.55± 2.40 | 0.22 ±1.89 | 3.5 ±1.9 | 1.6± 1.1 |  | 0.8± 1.9 |  |
| **LL to E line** | 6.76 ±2.83 | 0.98 ±2.06 | 1.40 ±2.23 | 3.5 ±2.1 | 2.8± 1.7 |  | 2.8± 2.2 |  |
| **LL to H line** |  | 1.32 ±1.17 | 1.37 ±1.35 |  |  | 0.5± 1 |  |  |
